# Trade‐offs and Synergies between Economic and Environmental Cocoa Farm Management Decisions

**DOI:** 10.1002/gch2.202400041

**Published:** 2024-07-10

**Authors:** Joseph Bandanaa, Isaac. K. Asante, Irene S. Egyir, Ted Y. Annang, Johan Blockeel, Anja Heidenreich, Irene Kadzere, Christian Schader

**Affiliations:** ^1^ Institute for Environment and Sanitation Studies University of Ghana Legon Box LG 209, Legon, Accra Ghana; ^2^ CSIR‐Plant Genetic Resources Research Institute (PGRRI) Eastern Region Bunso Box 7, Bunso Ghana; ^3^ Department of Plant and Environmental Biology University of Ghana Legon Box LG 55, Legon, Accra Ghana; ^4^ Department of Agricultural Economics and Agribusiness University of Ghana Legon Box LG 68, Legon, Accra Ghana; ^5^ Research Institute of Organic Agriculture (FiBL) Frick Ackerstrasse 113, CH‐5070 Frick Switzerland

**Keywords:** cocoa, farm management decisions, farming systems, organic, sustainability, trade‐offs

## Abstract

Optimizing sustainability among smallholder farms poses challenges due to inherent trade‐offs. In the study of organic and conventional cocoa smallholder farming in Ghana, 398 farms are assessed using the Food and Agriculture Organsation of the United Nations (FAO) Sustainability Assessment of Food and Agriculture systems (SAFA) Guidelines and Sustainability Monitoring and Assessment Routine (SMART)‐Farm Tool. Organic farming exhibited synergies in environmental aspects (e.g., soil quality, energy efficiency) and between biodiversity conservation and risk management. Conventional farming showed potential vulnerabilities, including trade‐offs with long‐range investments (e.g., chemical inputs) and species diversity. Both systems demand tailored approaches for short‐term economic and environmental sustainability, aligning with community‐wide long‐term goals. To mitigate trade‐offs in conventional farming, smallholders should adopt practices like material reuse, recycling, and recovery within their operations.

## Graphical abstract

Optimizing sustainability among smallholder farms poses challenges due to inherent trade‐offs. In the study of organic and conventional cocoa smallholder farming in Ghana, 398 farms are assessed using the FAO SAFA Guidelines and SMART‐Farm Tool. Organic farming exhibited synergies in environmental aspects (e.g., soil quality, energy efficiency) and between biodiversity conservation and risk management. Conventional farming showed potential vulnerabilities, including trade‐offs with long‐range investments (e.g., chemical inputs) and species diversity. Both systems demand tailored approaches for short‐term economic and environmental sustainability, aligning with community‐wide long‐term goals. To mitigate trade‐offs in conventional farming, smallholders should adopt practices like material reuse, recycling, and recovery within their operations.

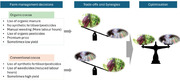

## 1 Introduction

Ghana is a significant player in the international cocoa market, being the second‐largest cocoa producer globally after Côte d“Ivoire and accounting for ≈20% of global production.^[^ ^1^ ^]^ Ghanaian cocoa is grown by small‐scale farmers, serving as a vital source of livelihood and contributing to farmers” social well‐being in terms of income and employment. Cocoa farming in Ghana employed over 600000 farmers in 2018.^[^ ^2^ ^]^

Cocoa in Ghana is predominantly grown using conventional practices characterized by the use of synthetic chemicals. Since 2007, these practices have been reinforced by several government interventions, including the Cocoa Disease and Pest Control Programme (CODAPEC) and the Cocoa Hi‐Tech program.^[^ ^3^, ^4^ ^]^ In the Atwima Mponua District, where the current study was conducted, projects promoting organic cocoa production and climate‐smart agriculture (CSA) have been introduced since 2010. Both organic and conventional farming systems in this district benefit from these interventions.^[^ ^5^ ^]^ The conventional cocoa production applies synthetic inputs and the organic often uses nature‐based inputs. Synthetic inputs include agrochemicals and laboratory‐based elements. Organic inputs are mainly frontier elements generated within the farm system, from plant and animal waste. However, the challenge remains whether these two farming systems meet the environmental and economic sustainability benefits.

The complexity of seeking sustainability and meeting multiple objectives in farming systems can arise when the benefits of one aspect result in negative consequences in another.^[^ ^6^ ^]^ Several authors have explored the challenge of meeting multiple sustainability objectives using a system thinking approach.^[^ ^7^, ^8^, ^9^, ^10^, ^11^ ^]^ These studies examine the interconnections and trade‐offs between different sustainability objectives in agriculture, considering a wide range of factors, including socio‐cultural valuation of ecosystem services, stakeholder engagement, and healthy diets from sustainable food systems.

For example, Fischer et al.^[^ ^7^ ^]^ found that the trade‐off between land sparing and land sharing is context‐dependent and that a combination of both approaches may be necessary to meet multiple sustainability objectives. Hodge and Adams^[^ ^8^ ^]^ emphasized the importance of using a system thinking approach to assess the trade‐offs and synergies between ecosystem services and bioenergy development. Iniesta‐Arandia et al.^[^ ^9^ ^]^ highlighted the need to understand the socio‐cultural values and drivers of change underlying the demand for ecosystem services to design effective policies for sustainable resource management. Reed et al.^[^ ^10^ ^]^ proposed a typology of stakeholder analysis methods to identify actors involved in natural resource management, their interests, and the power dynamics shaping decision‐making processes. Willett et al.^[^ ^11^ ^]^ argued that transforming the food system is necessary to achieve multiple sustainability objectives and proposed guidelines for healthy and sustainable diets considering environmental, social, and economic dimensions.

Farmers often face conflicting goals of increasing yield and protecting the environment due to their diverse roles as managers, workers, or community members. As farm managers, they may seek to increase cash flow by relying on pesticides and other productivity‐enhancing inputs, which can be harmful to the environment.^[^ ^12^ ^]^ As community members, they may conform to local norms and values.^[^ ^13^ ^]^ Due to these internal tensions, farmers may not implement a consistent strategy toward environmental protection, or their priorities may change over time relative to the membership and practices of a farming system. Trade‐off situations require making choices or managerial actions between alternatives that may not be achievable simultaneously.^[^ ^14^, ^15^, ^16^ ^]^

On the other hand, synergies are characterized by “win‐win strategies” between economic and environmental goals.^[^ ^17^ ^]^ For example, Bandanaa et al.^[^ ^18^ ^]^ examined the general sustainability performance of cocoa farming systems and found that conventional farms negatively impact soil health, water quality, and biodiversity. Additionally, they identified economic challenges faced by cocoa farmers, such as low productivity and lack of liquidity, which can lead to poverty and food insecurity. Their study highlights the importance of addressing trade‐offs and synergies between economic and environmental sustainability in cocoa farming to achieve more sustainable and equitable outcomes for farmers and the environment. The assertion that measures to improve the environmental sustainability of cocoa farming systems would also lead to economic benefits needs to be tested.

The objective of the current study is to identify the economic and environmental trade‐offs and synergies in organic and conventional cocoa farming systems for smallholder farm management decisions. Identifying these trade‐offs and synergies will inform farm management decisions, allowing policymakers and stakeholders to understand the hidden consequences of preferring one intervention or approach over another.^[^ ^19^ ^]^

The study on trade‐offs and synergies in organic and conventional cocoa smallholder farming in Ghana makes substantial contributions to the scientific literature on sustainable agriculture in several ways. First, by using the FAO SAFA Guidelines and the SMART‐Farm Tool, the study provides a comprehensive evaluation of sustainability. This approach considers environmental, social, economic, and governance dimensions, offering a balanced view of both organic and conventional farming systems, and promoting standardized and multidimensional evaluation methods in agricultural sustainability research.

The findings on environmental synergies in organic farming contribute to the understanding of the benefits of organic farming beyond yield metrics, emphasizing its role in enhancing ecosystem services and resilience. This can inform policy and decision‐making to support organic practices.

Furthermore, conventional farming showed potential vulnerabilities, particularly in terms of long‐range investments and species diversity. The reliance on synthetic chemical inputs can undermine long‐term sustainability by degrading soil health and reducing biodiversity. Identifying these vulnerabilities is crucial for developing interventions that can mitigate negative impacts.

Additionally, the study emphasizes that both organic and conventional systems require tailored approaches to achieve short‐term economic and environmental sustainability, while also aligning with long‐term community‐wide goals. This suggests that one‐size‐fits‐all solutions are insufficient. Policymakers and practitioners should consider local contexts and specific needs of farming communities to design effective sustainability strategies.

## 2 Experimental Section

### 2.1 Study Area

**Figure 1:**
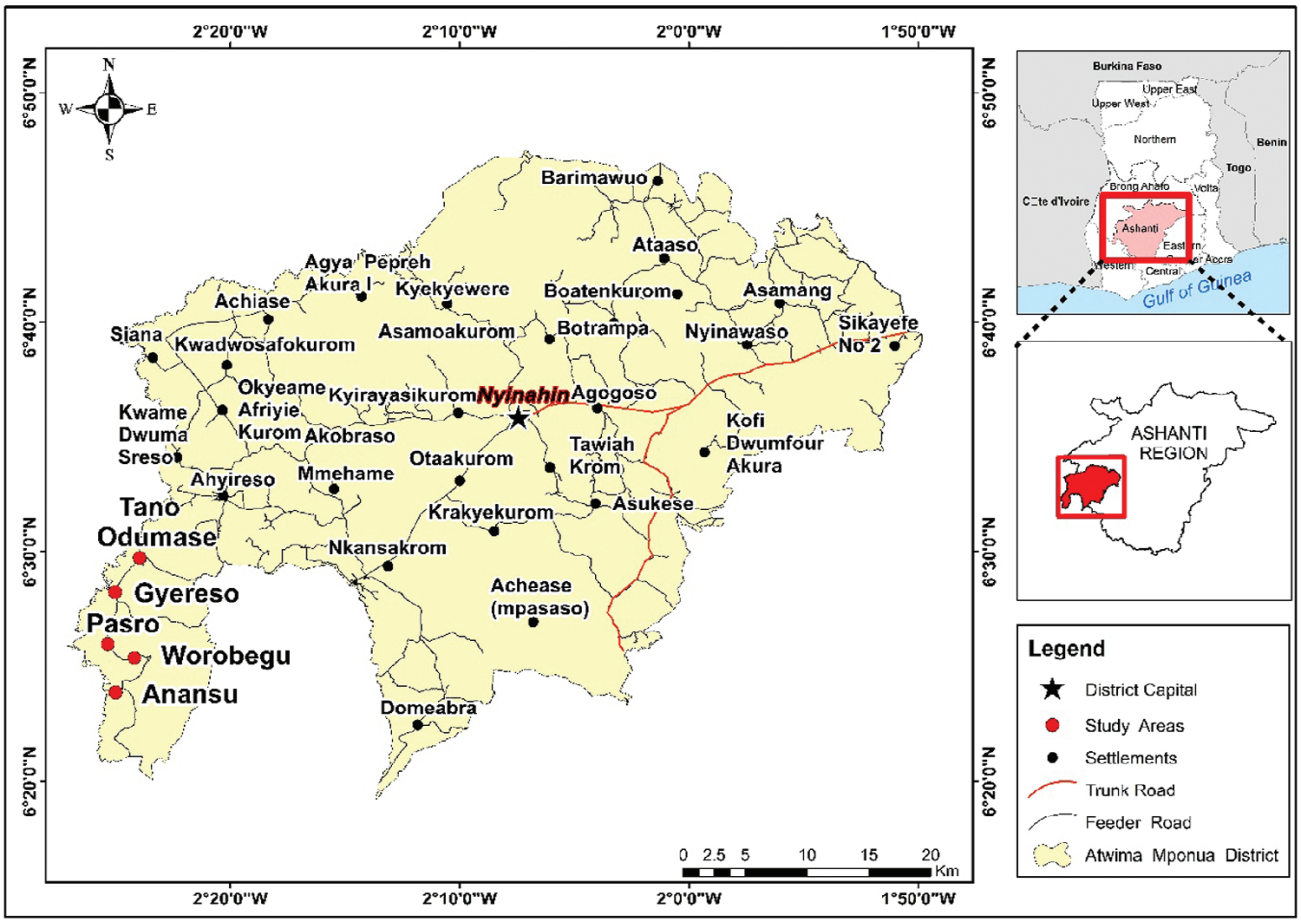
Map of the study area.

The study was conducted between November 2016 and February 2017 in five communities in the Atwima Mponua district, the south‐western part of the Ashanti Region in Ghana (see **Figure** **1**). Atwima Mponua District exemplifies an area where the organic farming system is promoted with other voluntary sustainability standards (VSS), for example, Rainforest Alliance (RA) since 2011.^[^ ^5^ ^]^ By default, the conventional cocoa farming system is expected in the case study communities as this is was the common practice, allowing this study to explore the two farming systems' economic and environmental trade‐offs and synergies within the same location.

The case study was characterized by moist semi‐deciduous forest vegetation and experiences a bimodal rainfall pattern ranging between 1250 and 1850 mm per annum, and average annual temperatures between 27.0 and 31.0 °C.^[^ ^20^ ^]^ About 66% of the economically active populations in the study site are engaged in agriculture,^[^ ^21^ ^]^ with smallholder cocoa farming as a significant activity. Cocoa is usually intercropped with plantain, annual crops, or other trees (both timber and non‐timber trees). The intercropping offers many agronomic benefits to smallholder farmers, including improving pollination, long‐term cocoa yield, and more profitability than from cocoa that is grown as a mono‐crop.^[^ ^22^ ^]^

### 2.2 Farm Characterization and Criteria for Defining Sample

#### 2.2.1 Farm Characterization

Organic and conventional farms were selected based on the inputs used. Farmers who had not applied any synthetic pesticides, inorganic fertilizers, and/or herbicides or who had applied only organic inputs (such as manures, composts, botanical pesticides, and organic fertilizers) from 2012 to 2017 were categorized as organic farmers. Through their Producer Group, all the organic cocoa farmers were registered under the internal control systems (ICS) and audited by the Control Union (CU), a tool for monitoring compliance within a producer group. Based on the categorization, 71 respondents as organic cocoa farmers, and 327 as conventional cocoa farmers were identified. Before the sustainability assessments began, these farmers had been participating, since 2014, in a comparative study on farm productivity and profitability.

#### 2.2.2 Criteria for Defining Sample

To explore the economic and environmental trade‐offs and synergies of organic and conventional cocoa farming systems, the Sustainability Monitoring and Assessment Routine (SMART)‐Farm Tool^[^ ^23^ ^]^ was applied using ten (10) trained enumerators. The enumerators visited and undertook a one‐time assessment of the selected farms using the SMART‐Farm Tool software. A face‐to‐face interview with the farm manager took an average of 2.5 h for an enumerator. In the study, farm managers were individuals who took care of the farms for or on behalf of the owner and could be the owner themselves. As part of the interview process, the enumerators visited the farmer's main fields, storage facilities, and/or livestock stables. Given that most indicator ratings are based on verbal information provided by the “farm manager”, factors undermining the credibility of the farmer's answers (e.g., fears and expectations) were addressed to avoid biased answers. At the start of the project, the communities, including farmers and local leaders, were sensitized on the data needs and objectives of the Organic Farming Systems for Africa (OFSA) project which included among other things, understanding the sustainable practices smallholder cocoa farmers employed in cocoa production.

#### 2.2.3 The SMART‐Farm Tool

Several tools are used to collect and analyze the sustainability of farms.^[^ ^24^, ^25^, ^26^ ^]^ These tools are categorized based on geographic applicability, primary purpose, thematic scope, and sustainability perspective.^[^ ^26^ ^]^ In terms of geographic applicability, these tools are either utilized in the developed world and, developing countries, or both. Most of the standard tools used in measuring and analyzing sustainability are the Committee on Sustainability Assessment (COSA), Response Inducing Sustainability Evaluation (RISE), SMART‐Farm Tool, and the Farm Sustainability Assessment (FSA).^[^ ^24^ ^]^ Most of the tools follow the traditional environmental, social, and economic thematic scopes. Few tools focus on only one of the three.^[^ ^27^, ^28^ ^]^ Other farm assessment tools include governance as the fourth thematic scope (e.g., SMART‐Farm Tool). Based on the primary purpose, geographic, and thematic scope, the Sustainability Monitoring and Assessment Routine (SMART‐Farm Tool) was selected for this current study.

**Figure 2:**
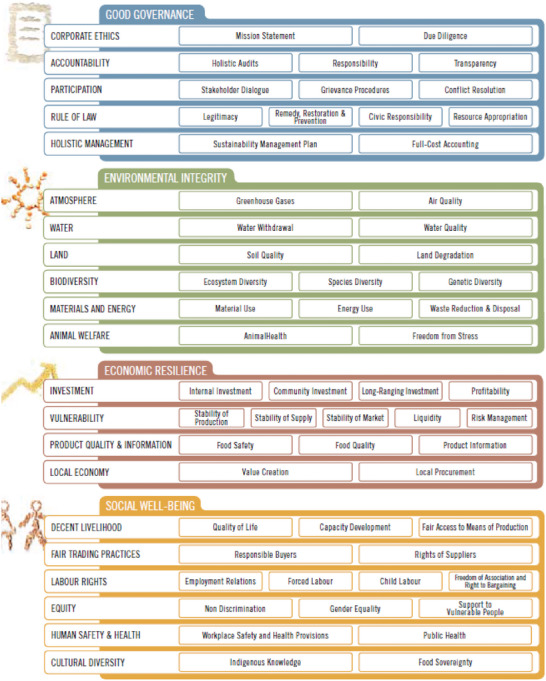
SAFA Sustainability dimension, themes, and sub‐themes (FAO, 2013).

The SMART‐Farm Tool, developed by an interdisciplinary team of experts, operationalizes the FAO's 2013 Sustainability Assessment of Food and Agriculture Systems (SAFA) Guidelines (see **Figure** **2**). As a result, it provides a fair system for assessing the sustainability of different farming systems. This is especially useful for organizations/individuals or enterprises to define sustainable agriculture differently^[^ ^29^ ^]^ and use different metrics to assess sustainable agricultural systems. The SAFA guidelines include four Sustainability Dimensions: “Environmental Integrity” (with six themes and fourteen sub‐themes), “Economic Resilience” (with four themes and fourteen sub‐themes), “Social Well‐Being” (with 6 themes and 16 sub‐themes), and “Good Governance” (with 5 Themes and 14 sub‐themes) as shown in Figure 2. The wide range of sustainability aspects defined by SAFA and measured by the SMART‐Farm Tool allows us to test and explore trade‐offs and synergies commonly assumed by farming systems in a credible manner.

### 2.3 Statistical Analysis

#### 2.3.1 Identifying Trade‐Offs and Synergies in Sustainability Sub‐Theme in Cocoa Farming Systems

A bivariate analysis was applied using Spearman's rank correlation to identify trade‐offs and synergies between economic and environmental sustainability sub‐themes (Table S1, Supporting Information). Negatively significant Spearman's rank correlation coefficients (*p* < 0.05) portrayed trade‐offs within and among the economic and environmental sustainability sub‐themes, while positively significant Spearman's correlation coefficients (*p* < 0.05) indicated synergies. The Spearman Rank Correlation was performed using the Corrplot package in RStudio v2021.09.403. Welch's *t*‐test was performed using RStudio v2021.09.403 to compare the statistical difference between farming systems for environmental and economic sustainability sub‐themes.

## 3 Results and Discussion

### 3.1 Farm Characteristics of Cocoa Smallholder Farming Systems

Results from the current study showed male dominance in cocoa production though there is no significant difference between organic and conventional.

The finding of male dominance in cocoa production, with no significant difference between organic and conventional farming systems, suggests that gender disparities in cocoa farming are widespread and not influenced by the type of farming practice. Similar findings have been reported in other regions; for example, Fasakin, Ajayi, and Olajide^[^ ^30^ ^]^ in Nigeria, and Kuhn, Tennhardt, and Lazzarini^[^ ^31^ ^]^ in Ecuador and Uganda. These studies stress that women face barriers in accessing resources such as credit, land, and labor, which ultimately deter their participation in production. Therefore, the need for gender‐sensitive interventions and policies to ensure equitable participation and benefits for women in cocoa farming is paramount.

The results showed that, on average, the farm size for an organic cocoa farmer was 2.3 hectares while that of conventional farmers was 3.2 hectares (**Table** **1**). Conventional farmers had a maximum farm size of 18 hectares, and that of organic was 6.9 hectares. The observed differences in average farm sizes between organic and conventional systems indicate potential scalability issues. Organic farms are generally smaller (average 2.3 hectares) compared to conventional farms (average 3.2 hectares), with the maximum farm size for conventional farms being significantly larger (18 hectares) than for organic farms (6.9 hectares). These findings align with a review conducted by Seufert and Ramankutty,^[^ ^32^ ^]^ which highlighted the prevalence of small farm sizes in organic production globally. This trend suggests that conventional farming systems may have a comparative advantage in terms of scalability, attracting farmers with larger land holdings who seek economic benefits that align with larger‐scale operations, as also observed by Bandanaa et al.^[^ ^18^ ^]^

**Table 1:** Farm Characteristics.

Variable	Organic (n = 71)	Conventional (n = 327)	P‐value
Gender			
Male	36 (50.7%)	186 (56.9%)	0.350
Female	35 (49.3%)	141 (43.1%)	
Education			
Literate	53 (74.6%)	248 (75.8%)	0.835
Illiterate	18 (25.4%)	79 (24.2%)	
Farm size (Ha)			
Mean	2.26	3.23	−0.000^a)^
Minimum	0.33	0.15	
Maximum	6.91	18.09	
Average hours spent per Hectare per activity per season			
Manual weeding	33.69	28.55	0.000^a)^
Pest management	3.62	4.01	0.014^c)^
Pruning	7.68	7.25	0.296
Harvesting	6.04	5.63	0.001^a)^
Pod breaking	6.88	5.95	0.000^a)^
Fermentation	6.51	7.59	‐0.003^a)^
Average hours spent on cocoa	12.04	10.05	0.000^a)^

^a)^ 1%;

^b)^ 5%;

^c)^ 10% significance; n = number

The average hours spent on the cultural practices to produce cocoa beans are also summarized in Table 1. Organic farmers spent on average 33.69 h per hectare per season performing manual weeding on their farm compared to conventional farmers, 28.55 h per hectare per season. This increased labor demand in organic farming is primarily due to the avoidance of chemical herbicides, which has implications for labor costs and labor availability. Addressing these labor demands is crucial, especially in regions with limited labor supply. The reduced time spent on weeding in conventional farms can be attributed to the use of herbicides, which, while reducing labor input, raise environmental and health concerns. These findings are consistent with a study by Seufert and Ramankutty,^[^ ^32^ ^]^ who found that organic farming is generally more labor‐intensive in terms of weeding. This trend is further supported by,^[^ ^33^ ^]^ who noted that organic farming systems require more labor than conventional systems, especially for labor‐intensive commodities, fruits, and tree crops. The increased labor requirement in organic farming may deter some farmers from adopting these practices unless compensated by higher market prices or other incentives.

Conventional farmers spent more hours managing pests and diseases (4.0 h) compared to organic 3.6 h (see Table 1). The need for regular application of chemical pesticides and fungicides in conventional farming likely reflects the increased time spent on pest and disease management. This practice has significant implications for the costs associated with chemical inputs and potential environmental impacts. Studies by McCoy and Frank,^[^ ^34^ ^]^ El‐Shafie,^[^ ^35^ ^]^ and Mohan et al.^[^ ^36^ ^]^ confirm the necessity of frequent pesticide applications in conventional farming systems. Organic farming, while spending slightly less time on pest and disease management, relies on labor‐intensive integrated pest management (IPM) practices, which include crop rotation, biological control, and mechanical cultivation. These methods necessitate specific knowledge and skills, highlighting the need for adequate training and support for organic farmers. Regulations restricting organic farmers to natural pesticides further emphasize the importance of comprehensive education in IPM techniques for sustainable pest control.

### 3.2 Interactions within Environmental and Economic Sustainability Goals for the Organic and Conventional Cocoa Farming Systems

**Figure 3:**
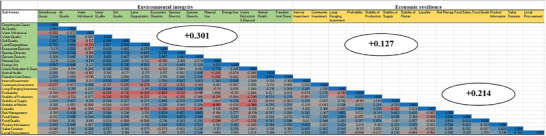
Trade‐offs and synergies among environmental and economic sustainability goals of organic cocoa farms.

**Figure 4:**
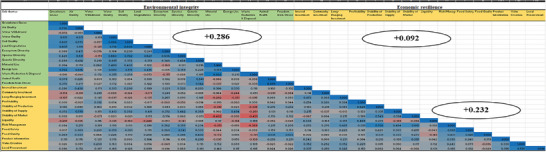
Trade‐offs and synergies among environmental and economic sustainability goals of conventional cocoa farms.

The interactions between the environmental goals for the organic and conventional farming systems are shown in **Figure** **3**. Of the possible 196 correlations, there were seventy‐seven (77) significant pairwise correlations within the environmental sub‐themes of the organic farming system (with 70 positives and 7 negatives). We found the soil quality sub‐theme (e.g., measures to prevent erosion) to be strongly positively correlated (*r* ≥ 0.5) with “water quality”, “energy use”, “air quality”, “species diversity”, lower “greenhouse gases”, and reduced “land degradation – no soil lost through degradation”. Water withdrawal was a trade‐off for greenhouse gases and land degradation (Figure 3). Seventy‐five significant pairwise correlations were found in the conventional farming system (71 positives, 4 negatives) (**Figure** **4**). Soil quality (e.g., measures to prevent erosion) strongly positively correlated (*r* ≥ 0.5) with “water quality” and “land degradation”. The ecosystem diversity (i.e., the functional integrity of the natural ecosystem and conservation) sub‐theme showed trade‐offs between waste reduction, disposal, and material use. For both farming systems, the study found synergies between implementing environmental goals like “animal health” and “freedom from stress”, “land degradation reduction measures”, and “soil quality”. More considerable synergies are observed within the environmental dimension (30.1%) for the organic farming system than conventional (28.6%), albeit with no statistical difference (**Table** **2**).

**Table 2:** Difference between farming systems for environmental and economic sustainability sub‐themes scores.

	Organic (n = 71)	Conventional (n = 327)	P‐value
*Environmental sub‐themes*			
Mean	56.55	53.69	6.374
Variance	30.1	28.6	
*Economic sub‐themes*			
Mean	44.60	42.83	0.002^a)^
Variance	21.4	23.2	

^a)^ Significant at 1%; n = number

The findings suggest that both farming systems exhibit significant environmental synergies, particularly concerning soil quality, water quality, and land degradation measures. The higher synergy rate in organic farming suggests that it may offer more integrated environmental benefits, supporting previous literature that highlights the ecological advantages of organic farming.^[^ ^32^, ^33^, ^37^ ^]^ However, the trade‐offs identified, such as those involving water withdrawal in organic systems and ecosystem diversity in conventional systems, underscore the complexity of achieving comprehensive sustainability. These trade‐offs align with studies indicating that optimizing one environmental goal can sometimes negatively impact another.^[^ ^7^, ^8^ ^]^

The results underscore the necessity for tailored approaches in implementing environmental goals to maximize synergies while mitigating trade‐offs. Policymakers and practitioners must consider these interactions to design effective sustainability strategies that address specific environmental, economic, and social contexts. The observed synergies and trade‐offs provide critical insights for developing balanced interventions that support both short‐term productivity and long‐term environmental health.

For the economic sustainability dimension, 64 significant pairwise correlations were found within the organic farming system (58 positives, 6 negatives) (Figure 3). Food safety had a strong synergistic relationship (*r* ≥ 0.5) with “risk management”, “long‐range investment”, “stability of supply”, and “food quality”. The stability of supply was a trade‐off for liquidity and profitability. Synergies were found for “profitability” and “liquidity” in the organic farming system. We found sixty‐five significant pairwise correlations (59 positives, 6 negatives) within the economic goals for the conventional farming system (Figure 4). Profitability had a strong synergistic relationship (*r* ≥ 0.5) with “stability of production”. Our study also showed synergies for “risk management” (e.g., land ownership) and “stability of market” (e.g., direct sales, no product returns and collective marketing) in the conventional farming system. For both farming systems, there were synergies between “risk management” (e.g., land ownership, climate adaptation measures) and “food safety” (certified usage of plant protection products and maintenance of food standards); and “value creation” and “local procurement”. We found significant synergies (Table 2) within the economic dimension (23.2%) for the conventional farming system compared to organic (21.4%), which emphasizes the influence of the conventional farming system on economic sustainability.

The findings regarding economic sustainability in cocoa farming systems clarify key aspects crucial for the sector's viability. The study's identification of numerous significant correlations within both organic and conventional farming systems underscores the intricate interplay of economic objectives inherent in cocoa production.^[^ ^38^ ^]^

In organic farming, the strong synergistic relationship observed between food safety and other economic factors like risk management, long‐range investment, stability of supply, and food quality underscores the interconnectedness of these goals.^[^ ^39^ ^]^ However, the identified trade‐off between stability of supply and liquidity/profitability suggests that maintaining consistent supply chains may compromise financial flexibility.^[^ ^32^ ^]^

Similarly, in conventional farming, profitability is closely tied to stability of production, highlighting the significance of consistent yields for economic resilience.^[^ ^40^ ^]^ Additionally, synergies between risk management and stability of market accentuate the pivotal role of market stability as a crucial risk mitigation strategy for conventional farmers.^[^ ^38^ ^]^

The study's findings also explain the importance of effective risk management practices, such as land ownership and climate adaptation measures, positively associated with both economic sustainability dimensions in both farming systems. Furthermore, the observed positive relationship between food safety and economic factors in the organic farming system emphasizes the necessity for holistic approaches to economic sustainability, considering multiple objectives simultaneously.^[^ ^41^ ^]^

Overall, the significant synergies identified within the economic dimension of the conventional farming system compared to organic farming shows the predominant influence of conventional practices on economic sustainability in cocoa production.^[^ ^38^ ^]^ This emphasizes the need for tailored interventions and support mechanisms to enhance economic sustainability across both organic and conventional cocoa farming systems.^[^ ^39^ ^]^

### 3.3 Interactions between Environmental and Economic Sustainability Goals for the Organic and Conventional Cocoa Farming Systems

The results revealed 137 significant pairwise correlations between environmental and economic goals for the organic farming system (106 positives, 31 negatives) (Figure 3). In the organic farming system, biodiversity (species diversity, genetic diversity, and ecosystem diversity) showed synergies with long‐range investment and risk management. Water quality showed a synergistic relationship with food safety, product information and internal investment. The study found “stability of market” to have a trade‐off with “material use” and “waste reduction and disposal”. “Internal investment” (i.e., continuous investment to enhance sustainability) in the organic farming system shows a synergistic relationship with “energy use”, soil quality, and lower “greenhouse gases”. Figure 4 shows 126 significant pairwise correlations between environmental and economic goals for the conventional farming system (96 positives, 30 negatives). “Freedom from stress” for animals (including chicken and ruminants) showed synergistic relations with “value creation” and “local procurement”. The study found “species diversity”, “land degradation” and “energy use” as key environmental goals negatively related (trade‐off) with economic goals such as liquidity, lack of community investment (e.g., sustainability training and management of riparian stripes), and long‐range investment (e.g., plant protection, soil improvement). “Species diversity” was negatively related (trade‐off) to “value creation”, “local procurement”, “long‐range investment”, and “product information”. Land degradation was negatively related (trade‐off) to “value creation”, “local procurement”, “long‐range investment”, and “product information”. We find major synergies between environmental and economic (12.7%) organic sub‐themes (Figure 3) compared to conventional sub‐themes (9.2%) (Figure 4).

The results regarding the interactions between environmental and economic sustainability goals in cocoa farming systems are significant and multifaceted. In the organic farming system, numerous significant correlations between environmental and economic goals were identified, highlighting the integrated nature of these dimensions.^[^ ^42^ ^]^ For instance, investing in biodiversity conservation not only enhances farm resilience but also contributes to long‐term economic stability. Biodiversity conservation, a key environmental goal in organic farming, demonstrated synergistic relationships with long‐range investment and risk management, aligning with previous research emphasizing the importance of biodiversity in enhancing farm resilience.^[^ ^43^ ^]^ Additionally, internal investment aimed at enhancing sustainability, such as soil improvement practices, is closely linked to economic benefits like energy efficiency and reduced greenhouse gas emissions, emphasizing the interconnectedness of economic and environmental objectives in organic farming.^[^ ^44^ ^]^

Conversely, in the conventional cocoa farming system, trade‐offs were observed between environmental and economic goals. For example, the use of chemical inputs may boost short‐term productivity but can have negative effects on species diversity and soil health, ultimately impacting long‐term sustainability. This suggests that certain economic activities in conventional farming may compromise environmental goals. These examples are cited in studies including Bandanaa et al.^[^ ^45^ ^]^ and Walker & Salt.^[^ ^42^ ^]^

Both organic and conventional cocoa farming systems exhibit trade‐offs and synergies between environmental and economic dimensions. However, the organic farming system demonstrated more synergies, indicating its potential for achieving greater alignment between economic resilience and environmental sustainability (Figures 3 and 4). This underscores the importance of considering farming system‐specific contexts and objectives in designing sustainable cocoa production strategies.^[^ ^42^ ^]^

### 3.4 Trade‐offs and Synergies in Cocoa Farming Systems and the Implication for Farm Management Decisions

Using the SMART‐Farm Tool, we explored trade‐offs and synergies in cocoa farming systems, identifying the relationships between desirable but competing economic and environmental goals. Using the SMART‐Farm Tool based on the globally valid SAFA guidelines, the study used an actor‐oriented approach to ask farmers/farm managers about their environmental and economic sustainability goals. Since trade‐offs and synergies differ between farming systems, this study examined the differences between organic and conventional cocoa farming systems. This study identified synergies in organic and conventional cocoa systems that would meet production's economic and environmental benefits.

The two farming systems place different emphases on sustainability. To achieve these sustainability goals, a combination of strategies and practices that will ensure the goals/sub‐themes are met should be encouraged in each system. To achieve biodiversity (species diversity, genetic diversity, and ecosystem diversity) in the conventional cocoa farming system, synthetic chemical use, particularly herbicides, should be limited.^[^ ^45^ ^]^ Rodenburg et al.^[^ ^46^ ^]^ observed that uncontrolled herbicide use had a negative relation with soil quality and livestock health in Sub‐Saharan Africa. Since these goals/sub‐themes themselves were mutually exclusive, trade‐offs occurred whenever sustainability goals/sub‐themes competed. Particularly, farmers in the conventional cocoa farming system are unaware of the consequences or lacked direct experience.^[^ ^47^, ^48^ ^]^

The high synergy between economic and environmental sub‐themes, particularly in the organic farming system, points to one major contextual factor – organic farmers receive training and are made aware of economic opportunities and environmental conservation from state and non‐state actors along the cocoa value chain. On the other hand, conventional cocoa farmers receive extension services and training from mostly the state actor, the Cocoa Health, and Extension Division (CHED) of the Ghana Cocoa Board. The emphasis on environmental conservation is predominantly more in organic training compared to conventional.

## 4 Limitations

The SMART‐Farm Tool provides a standardized multi‐criteria evaluation technique for transparently comparing farms across various agricultural systems and geographical locations and assessing them against the FAO‐SAFA Guidelines. Like most farm assessment tools, the SMART‐Farm Tool has limitations and the potential for interpretation error. For example, in terms of accuracy to particular subthemes^[^ ^23^ ^]^ and the uncertainties around the indicator weights.^[^ ^18^ ^]^ Thus, performing detailed trade‐offs and synergy analysis is somewhat limiting in terms of its provision of in‐depth quantitative data as the scoring for indicators and sub‐themes ranges from 0–100%.

## 5 Conclusion and Recommendations

The analysis of interactions between environmental and economic sustainability goals shows the potentially greater vulnerability of the conventional farming system to trade‐offs compared to organic practices. Liquidity and long‐range investment, notably pesticide use, in conventional farming, can lead to a decline in species diversity. While organic farmers may manage trade‐offs more effectively, their practices, involving long‐term commitments to soil and water conservation, present challenges. Tailored support mechanisms are essential for both farming systems to achieve short‐term economic and environmental sustainability goals while contributing to long‐term community objectives. Synergistic practices addressing economic and environmental dimensions can aid in this endeavor. Continuous investment in technologies enhancing energy use efficiency, soil quality, and greenhouse gas reduction is imperative for conventional farming. Additionally, promoting practices ensuring the reuse, recycling, and recovery of materials is vital for reducing environmental and economic trade‐offs. Policymakers should implement support mechanisms tailored to each farming system's needs, including incentives, resources for conservation, and capacity‐building programs. These efforts will foster sustainable cocoa farming practices that benefit both farmers and the environment.

## Conflict of Interest

The authors declare no conflict of interest.

## Data Availability

The data that support the findings of this study are available from the corresponding author upon reasonable request.
